# Cancer among children of parents with autoimmune diseases

**DOI:** 10.1054/bjoc.1999.1104

**Published:** 2000-03-06

**Authors:** L Mellemkjær, F Alexander, J H Olsen

**Affiliations:** Institute of Cancer Epidemiology, Danish Cancer Society, Strandboulevarden 49, Copenhagen Ø, 2100, Denmark; Department of Public Health Sciences, The University of Edinburgh, Medical School, Teviot Place, Edinburgh, EH8 9AG, UK

**Keywords:** childhood lymphoma, childhood leukaemia, parental autoimmune disease

## Abstract

Many different aetiologies for childhood cancer have been suggested, but few are well established. One is that parental autoimmune disease is linked with susceptibility for haematopoietic malignancies in their offspring during childhood. The present study is the first to investigate this hypothesis using a follow-up design. A cohort of 53 811 children of more than 36 000 patients diagnosed with a systemic, organ-specific or suspected autoimmune disease were followed up for cancer incidence in the Danish Cancer Registry during 1968–1993. The parents were identified through the National Registry of Patients, while their children were traced in the Central Population Register. Cancer incidence among the offspring was compared with that in the corresponding childhood population of Denmark. In total, 115 cancers were observed among children aged 0–19 years, yielding a non-significant standardized incidence ratio of 1.07. Lymphomas contributed 21 cases to the overall number of tumours, 60% more than expected (95% confidence interval (CI) 1.0–2.4); leukaemia contributed 37 cases representing an excess of 30% (95% CI 0.9–1.8). Our results give some support to the hypothesis that parental autoimmune disease is associated with childhood lymphoma and leukaemia. © 2000 Cancer Research Campaign


British Journal of Cancer (2000) **82**(7), 1353–1357
© 2000 Cancer Research Campaign
DOI: 10.1054/ bjoc.1999.1104, available online at http://www.idealibrary.com on IDEAL®

### Cancer among children of parents with autoimmune diseases

**L Mellemkjær[1], F Alexander[2] and JH Olsen[1]**

[1]Institute of Cancer Epidemiology, Danish Cancer Society, Strandboulevarden 49, 2100 Copenhagen Ø, Denmark; [2]Department of Public Health Sciences, The University of Edinburgh, Medical School, Teviot Place, Edinburgh EH8 9AG, UK

**Summary** Many different aetiologies for childhood cancer have been suggested, but few are well established. One is that parental autoimmune disease is linked with susceptibility for haematopoietic malignancies in their offspring during childhood. The present study is the first to investigate this hypothesis using a follow-up design. A cohort of 53 811 children of more than 36 000 patients diagnosed with a systemic, organ-specific or suspected autoimmune disease were followed up for cancer incidence in the Danish Cancer Registry during 1968–1993. The parents were identified through the National Registry of Patients, while their children were traced in the Central Population Register. Cancer incidence among the offspring was compared with that in the corresponding childhood population of Denmark. In total, 115 cancers were observed among children aged 0–19 years, yielding a non-significant standardized incidence ratio of 1.07. Lymphomas contributed 21 cases to the overall number of tumours, 60% more than expected (95% confidence interval (CI) 1.0–2.4); leukaemia contributed 37 cases representing an excess of 30% (95% CI 0.9–1.8). Our results give some support to the hypothesis that parental autoimmune disease is associated with childhood lymphoma and leukaemia. © 2000 Cancer Research Campaign

**Keywords**: childhood lymphoma; childhood leukaemia; parental autoimmune disease

The hypothesis that children whose families suffer from autoimmune disease have increased risk of haematopoietic malignancies was first raised in 1979 (Till et al, 1979). Since then, a few small epidemiological studies have tested the hypothesis (Till et al, 1979; McKinney et al, 1987; Woods et al, 1987; Buckley et al, 1989, 1994; Wen et al, 1998), providing some support for the association. Further support comes from evidence that inherited susceptibility to Hodgkin's disease, and possibly, also acute lymphocytic lymphoma (ALL), is linked to HLA class II (Bodmer et al, 1989; Tonks et al, 1992; Klitz et al, 1994; Taylor et al, 1995, 1996, 1998) as is the case for most autoimmune diseases (Deodhar, 1992). Best available epidemiological evidence suggests that childhood leukaemias and lymphomas arises as a rare sequela of certain patterns of exposure to infection (Doll, 1999). For the dominant subtype of ALL, common ALL, it has been proposed that leukaemia follows an overly florid immune response (Greaves, 1997), as is believed to occur in autoimmune disease.

In this nationwide study from Denmark, the incidences of malignant lymphoma, leukaemia and other childhood cancers were evaluated in a large cohort of offspring of patients with autoimmune disease ascertained through a hospitalization register and compared with that of the general childhood population.

### MATERIALS AND METHODS

The study population comprised the children of 36 654 patients born after 1 January 1930 and registered in the Danish National Registry of Patients, primarily during 1977–1993 with one or more of the autoimmune diseases shown in Table 1. The National Registry of Patients contains information about more than 99% of hospitalizations in non-psychiatric hospitals, with registration of the personal identification number, dates of hospitalization and up to 20 discharge diagnoses coded according to a Danish modified version of ICD-8 (Danish National Board of Health, 1976). The autoimmune diseases included cover a wide spectrum of diseases, which may be divided into three main categories: systemic (28% of patients), organ-specific (31%) and suspected autoimmune diseases (42%) (Table 1).

The Danish Central Population Register (CPR) was established on 1 April 1968, when all residents of Denmark were assigned a unique personal identification number which includes the sex and date of birth of the individual. Since that date, the identification number has been given to all children at birth. The Register keeps track of all persons ever assigned an identification number in respect of vital status and addresses; in addition, the Register includes a cross-reference between children and parents alive on 1 April 1968 who were registered at the same address. Because of the latter condition, we chose to restrict the study population to children of parents born after 1930. We were thus able to trace 56 052 children in the CPR, corresponding to 1.5 children per parent. Of these, we excluded 197 children from Greenland, 2043 children who were born after 1993 and one child whose 'parent' was only 10 years old at the time of the child's birth, thus leaving 53 811 children for follow-up for cancer. For these children, the fathers with autoimmune disease were on average 28.4 years old when the child was born, while the mothers with autoimmune disease were on average 25.7 years old at the time of the birth of the child.

The offspring were followed for childhood cancer from their date of birth or the date for establishment of the CPR (1 April 1968), whichever occurred latest, until the date of cancer diag-

*Received 2 August 1999*
*Revised 14 October 1999*
*Accepted 14 October 1999*

*Correspondence to:* L Mellemkjær

**Table 1** Numbers of patients with systemic, organ-specific and suspected autoimmune disease and the number of offspring

| Autoimmune disease[a] | ICD-8 | Period in the NRP | No. of patients | No. of offspring |
|---|---|---|---|---|
| All | | | 36 654 | 53 811 |
| Systemic autoimmune diseases[b] | | | 10 159 | 15 487 |
| Systemic scleroderma | 734.0 | 1977–1993 | 376 | 573 |
| Rheumatoid arthritis | 712.0–712.3, 712.5 | 1977–1993 | 8 012 | 12 294 |
| Polymyositis/dermatomyositis | 716.0–716.1 | 1977–1993 | 348 | 511 |
| Systemic lupus erythematosus | 734.1 | 1977–1993 | 1 163 | 1 588 |
| Sjögren's syndrome | 734.90[c] | 1977–1993 | 260 | 521 |
| Organ-specific autoimmune diseases[b] | | | 11 225 | 14 396 |
| Grave's disease | 242.00[c] | 1977–1993 | 319 | 602 |
| Hashimoto's thyroiditis | 245.03[c] | 1977–1993 | 326 | 631 |
| Juvenile diabetes mellitus | 249.0 | 1987–1989 | 8 652 | 10 416 |
| Pernicious anaemia | 281.0 | 1977–1989 | 427 | 680 |
| Autoimmune haemolytic anaemia | 283.90[c] | 1977–1993 | 103 | 99 |
| Idiopathic thrombocytopenic purpura | 287.10[c] | 1977–1989 | 452 | 487 |
| Primary biliary cirrhosis | 571.90[c] | 1977–1989 | 161 | 254 |
| Localized scleroderma | 701.0 | 1977–1993 | 365 | 600 |
| Myasthenia gravis | 733.0 | 1977–1993 | 420 | 627 |
| Suspected autoimmune diseases[d] | | | 15 270 | 23 928 |
| Sarcoidosis | 135.9 | 1977–1993 | 3 412 | 5 802 |
| Multiple sclerosis | 340.0 | 1977–1993 | 4 809 | 7 457 |
| Amyotrophic lateral sclerosis | 348.0 | 1977–1993 | 280 | 506 |
| Periarteritis nodosa | 446.0 | 1977–1993 | 237 | 365 |
| Wegener's granulomatosis | 446.2 | 1977–1993 | 152 | 206 |
| Temporal arteritis and polymyalgia arterica | 446.3 | 1977–1993 | 568 | 1 079 |
| Ulcerative colitis | 563.1 | 1977–1989 | 4 549 | 6 545 |
| Ankylosing spondylitis | 712.4 | 1977–1993 | 1 263 | 1 968 |

NRP, National Registry of Patients. [a]In case of overlap between the main groups, the patient was counted in the group of highest priority (1, systemic; 2, organ-specific; 3, suspected). In case of overlap within a main group, the disease with the earliest date of hospitalization was counted. [b]The classification of diseases into systemic and organ-specific autoimmune diseases is slightly modified after Theofilopoulos (1993). [c]Specific for the Danish modified version of ICD-8. [d]Termed suspected autoimmune diseases according to Cid et al, 1989; Savage and Lockwood, 1990; Lakomek et al, 1991; Forgacs, 1993; Lucchinetti and Rodriguez, 1997; Moller, 1997; Jackson and Bryan, 1998.

nosis, the age of 20, date of death, date of emigration or 31 December 1993. Cases of cancer occurring during the follow-up were ascertained by linkage to the Danish Cancer Registry, which receives notifications from clinicians whenever a malignant neoplasm, a benign tumour in the nervous system or a papilloma of the urinary tract is diagnosed or when changes in the initial diagnosis occur. All tumours are coded according to a revised version of ICD-7 (Danish National Board of Health, 1998), and since 1978 also according to ICD-O (World Health Organization, 1976). We grouped tumours diagnosed during childhood by a classification scheme for childhood cancers prepared by the International Agency for Research on Cancer (Birch and Marsden, 1987), which is based on ICD-O. For childhood cancers registered before 1978, the original diagnosis given on the notification forms has been reviewed and an ICD-O code assigned to each tumour, as described previously (Brown et al, 1989), so that the childhood cancer classification scheme could be used throughout the study period, 1968–1993.

The expected numbers of tumours were calculated from accumulated person-years and national childhood cancer incidence rates for each sex and for specific age groups (< 1, 1–4, 5–9, 10–14 and 15–19 years) and 5-year calendar periods. As a measure of the relative risk, standardized incidence ratios (SIR) were computed by dividing the expected number into the observed number; 95% confidence intervals (CI) were calculated assuming a Poisson distribution of the observed numbers by Byar's approximation (Rothman and Boice, 1979).

### RESULTS

The offspring cohort consisted of 28 148 (52%) males and 25 663 (48%) females. The date of entry was equal to the date of birth for 61% of the children, while 32% entered the cohort at the ages of 1–10 years. Of the offspring, 188 were themselves reported to have an autoimmune disease in the files of the National Registry of Patients; none of these had a diagnosis of cancer during childhood.

During the childhood follow-up period that continued until the age of 20, 750 581 person-years were accrued, with an average follow-up of 13.9 years. A total of 115 cancers were observed, which resulted in a SIR of 1.07 (95% CI 0.89–1.29) (Table 2). The risk for lymphomas was increased by 1.6-fold on the basis of 21 observed cases (95% CI 1.0–2.4); these included four cases of Burkitt's lymphoma (SIR 3.1; 95% CI 0.8–7.9). There were 37 cases of leukaemia, whereas 28.2 were expected, to yield a SIR of 1.3 (95% CI 0.9–1.8). Among the specific types of leukaemia, the largest increase in risk was seen for acute non-lymphocytic leukaemia (ANLL: SIR 1.9; 95% CI 0.9–3.7), whereas no excess risk was observed for ALL (SIR 1.1; 95% CI 0.7–1.7). There was no excess risk for any other type of childhood cancer.

There was no apparent difference between boys and girls in the risk for either lymphoma or leukaemia (Table 3). When age at cancer diagnosis was considered, the excess of lymphomas was most pronounced in children aged 1–14 years, while for leukaemias there was an excess of infant leukaemia (< 1 year) and

 *© 2000 Cancer Research Campaign*

**Table 2** Standardized incidence ratios (SIR) of childhood cancers among offspring of patients with autoimmune diseases followed up to their 20th birthday

| Cancer site | Obs | Exp | SIR | 95% CI |
|---|---|---|---|---|
| All malignant neoplasms | 115 | 107.1 | 1.07 | 0.89–1.29 |
| Lymphomas and other reticuloendothelial neoplasms | 21 | 13.6 | 1.6 | 1.0–2.4 |
| Hodgkin's disease | 8 | 6.2 | 1.3 | 0.6–2.5 |
| Non-Hodgkin's lymphomas | 7 | 4.5 | 1.5 | 0.6–3.2 |
| Burkitt's lymphoma | 4 | 1.3 | 3.1 | 0.8–7.9 |
| Unspecified lymphoma | 2 | 1.2 | 1.6 | 0.2–5.8 |
| Leukaemias | 37 | 28.2 | 1.3 | 0.9–1.8 |
| Acute lymphocytic | 22 | 19.8 | 1.1 | 0.7–1.7 |
| Acute non-lymphocytic | 9 | 4.6 | 1.9 | 0.9–3.7 |
| Chronic myelocytic | 1 | 0.8 | 1.3 | 0.0–7.2 |
| Other and unspecified | 5 | 3.0 | 1.7 | 0.5–3.9 |
| Other types of neoplasms | 57 | 65.3 | 0.9 | 0.7–1.1 |
| Central nervous system | 22 | 25.6 | 0.9 | 0.5–1.3 |
| Sympathetic nervous system | 5 | 5.3 | 0.9 | 0.3–2.2 |
| Retinoblastomas | 1 | 2.0 | 0.5 | 0.0–2.7 |
| Renal tumours | 1 | 4.9 | 0.2 | 0.0–1.1 |
| Hepatic tumours | 1 | 0.8 | 1.2 | 0.0–6.6 |
| Malignant bone tumours | 2 | 5.4 | 0.4 | 0.0–1.3 |
| Soft-tissue sarcomas | 8 | 5.9 | 1.4 | 0.6–2.7 |
| Germ-cell, trophoblastic and other gonadal | 8 | 7.1 | 1.1 | 0.5–2.2 |
| Carcinomas, other and unspecified malignant | 9 | 9.7 | 0.9 | 0.4–1.8 |

CI, confidence interval.

**Table 3** Risks for leukaemia and lymphoma in childhood among offspring of patients with autoimmune diseases according to sex, age at time of diagnosis of cancer, sex of the parent and type of autoimmune disease

| | No. of offspring | Lymphomas | | | | Leukaemias | | | | Combined | |
|---|---|---|---|---|---|---|---|---|---|---|---|
| Variable | | Obs | Exp | SIR | 95% CI | Obs | Exp | SIR | 95% CI | SIR | 95% CI |
| Sex | | | | | | | | | | | |
| Male | 28 148 | 13 | 9.2 | 1.4 | 0.8–2.4 | 20 | 16.3 | 1.2 | 0.8–1.9 | 1.3 | 0.9–1.8 |
| Female | 25 663 | 8 | 4.4 | 1.8 | 0.8–3.6 | 17 | 11.9 | 1.4 | 0.8–2.3 | 1.5 | 1.0–2.3 |
| Age at diagnosis of cancer | | | | | | | | | | | |
| Infant lymphoma/leukaemia (< 1 year) | | 0 | 0.2 | – | – | 4 | 1.5 | 2.8 | 0.7–7.0 | – | – |
| Childhood lymphoma (1–14 years) | | 13 | 7.0 | 1.9 | 1.0–3.2 | – | – | – | – | – | – |
| Adolescent lymphoma (15–19 years) | | 8 | 6.4 | 1.3 | 0.5–2.5 | – | – | – | – | – | – |
| Peak ALL (1–4 years) | | – | – | – | – | 9 | 8.7 | 1.0 | 0.5–2.0 | – | – |
| Older ALL (5–19 years) | | – | – | – | – | 12 | 10.6 | 1.1 | 0.6–2.0 | – | – |
| Older AML and other (1–19 years) | | – | – | – | – | 12 | 7.6 | 1.6 | 0.8–2.8 | – | – |
| Parent with autoimmune disease | | | | | | | | | | | |
| Father | 22 218 | 10 | 5.6 | 1.8 | 0.9–3.3 | 18 | 12.1 | 1.5 | 0.9–2.4 | 1.6 | 1.1–2.3 |
| Mother | 31 248 | 11 | 7.9 | 1.4 | 0.7–2.5 | 19 | 16.0 | 1.2 | 0.7–1.9 | 1.3 | 0.9–1.8 |
| Both | 345 | 0 | 0.1 | – | – | 0 | 0.2 | – | – | – | |
| Type of autoimmune disease | | | | | | | | | | | |
| Systemic | 15 487 | 6[a] | 4.2 | 1.4 | 0.5–3.1 | 12[a] | 7.9 | 1.5 | 0.8–2.6 | 1.5 | 0.9–2.3 |
| Father | 4 340[b] | 1 | 1.2 | 0.8 | 0.0–4.7 | 3 | 2.3 | 1.3 | 0.3–3.8 | 1.1 | 0.3–2.9 |
| Mother | 10 989[b] | 5 | 3.0 | 1.7 | 0.5–3.9 | 9 | 5.5 | 1.6 | 0.7–3.1 | 1.7 | 0.9–2.8 |
| Organ-specific | 14 396 | 4[c] | 3.4 | 1.2 | 0.3–3.0 | 9[c] | 7.4 | 1.2 | 0.6–2.3 | 1.2 | 0.6–2.1 |
| Father | 6 765[b] | 3 | 1.7 | 1.8 | 0.4–5.3 | 8 | 3.6 | 2.2 | 1.0–4.3 | 2.1 | 1.0–3.7 |
| Mother | 7 518[b] | 1 | 1.7 | 0.6 | 0.0–3.2 | 1 | 3.7 | 0.3 | 0.0–1.5 | 0.4 | 0.0–1.3 |
| Suspected | 23 928 | 11[d] | 6.0 | 1.9 | 0.9–3.3 | 16[e] | 12.9 | 1.2 | 0.7–2.0 | 1.4 | 0.9–2.1 |
| Father | 11 113[b] | 6 | 2.7 | 2.2 | 0.8–4.8 | 7 | 6.1 | 1.2 | 0.5–2.4 | 1.5 | 0.8–2.5 |
| Mother | 12 741[b] | 5 | 3.2 | 1.6 | 0.5–3.6 | 9 | 6.8 | 1.3 | 0.6–2.5 | 1.4 | 0.8–2.4 |

ALL, acute lymphocytic leukaemia; AML, acute myelocytic leukaemia. [a]All parents had rheumatoid arthritis. [b]Children whose parents both had an autoimmune disease are not included. [c]All parents had diabetes mellitus. [d]Parents had sarcoidosis ($n$ = 3), multiple sclerosis ($n$ = 4), ulcerative colitis ($n$ = 1) and ankylosing spondylitis ($n$ = 3). [e]Parents had sarcoidosis ($n$ = 3), multiple sclerosis ($n$ = 5), amyothrophic lateral sclerosis ($n$ = 2), polymyalgia arterica ($n$ = 1), ulcerative colitis ($n$ = 4) and ankylosing spondylitis ($n$ = 1).

of acute myelocytic and other leukaemia at the ages 1–19 years. Excess risks were seen for both lymphoma and leukaemia among children of fathers and of mothers with autoimmune diseases, while no cases were observed among the 345 children who had both a father and a mother with a relevant disease. The fathers were on average 30.0 years of age and mothers 27.0 years when

© 2000 Cancer Research Campaign the child with leukaemia or lymphoma was born. For all 58 cases of leukaemia and lymphoma, the parents were hospitalized for the autoimmune disease subsequent to the birth of the child, but we do not have a date of diagnosis of the autoimmune disease.

When considering the main type of autoimmune disease in the parent, there was no statistical significant difference in risk for either lymphoma or leukaemia, although the risk for lymphomas tended to be higher among children of parents with suspected autoimmune disease than among those of parents with systemic or organ-specific disease (Table 3). The analysis of type of autoimmune disease was further stratified according to whether the mother or father had the disease in question. This analysis showed that the increased risk for lymphoma and leukaemia was associated with maternal systemic disease and paternal organ-specific disease and maternal or paternal suspected disease. The parental systemic disease was rheumatoid arthritis for all 18 children with lymphoma or leukaemia, the parental organ-specific disease was diabetes mellitus for all 13 case children, while several different suspected diseases were diagnosed in parents of the remaining 27 case children (multiple sclerosis $n = 9$, sarcoidosis $n = 6$, ulcerative colitis $n = 5$, ankylosing spondylitis $n = 4$, amyotrophic lateral sclerosis $n = 2$, polymyalgia arterica $n = 1$).

### DISCUSSION

In this large cohort of children of parents with autoimmune disease, we found a moderately increased risk for childhood lymphomas and leukaemia of borderline statistical significance; it accords, however, with the a priori hypothesis of an association between autoimmune disease and childhood lymphoma and leukaemia in offspring; these haematopoietic cancers were the only types of childhood cancer out of many considered for which an association was seen.

The background for investigating an association between parental autoimmune disease and childhood haematopoietic cancer is the convergence of aetiological hypotheses for autoimmune disease and childhood leukaemia or lymphoma. Both disease entities may arise as a rare response to exposure to one or more infectious agents (Deodhar, 1992; Fox et al, 1992; Doll, 1999), the probability of this response being influenced by host factors which include age and inherited factors. As infectious aetiology may also be relevant for adult non-Hodgkin's lymphoma, the hypothesis may also be relevant to certain autoimmune diseases and non-Hodgkin's lymphoma within the same individual (Kassan et al, 1978; Gridley et al, 1993; Mellemkjær et al, 1997).

Previous studies of the hypothesis are case–control studies, potentially subject to recall bias. They have reported associations of ALL with familial autoimmune disease (Till et al, 1979), maternal multiple sclerosis (Buckley et al, 1989) and familial ulcerative colitis (Buckley et al, 1994). A further study showed an association between ALL and maternal antinuclear antibodies, though not overt autoimmune disease (Woods et al, 1987). Absence of an association has been reported for acute myeloid leukaemia with familial autoimmune disease (Till et al, 1979), parental multiple sclerosis with acute non-lymphoblastic leukaemia and lymphomas (Buckley et al, 1989), parental connective tissue diseases (of which some are autoimmune) with leukaemia or non-Hodgkin's lymphoma (McKinney et al, 1987), and infant leukaemia (which has distinct cytogenic and presumed aetiological characteristics (Greaves, 1997)) with parental autoimmune disease (Wen et al, 1998). Overall, these studies are not adequate for an evaluation, since most of them only included up to a few hundred cases of leukaemia and lymphoma, and definitions of autoimmune diseases among family members differed widely as did case groups.

The present study is, we believe, the first to evaluate the hypothesis in a follow-up design. The possibility of identifying index persons and their offspring through computerized, nationwide registers meant that an extraordinarily large study population of offspring could be collected. Another advantage of registry data in comparison with data used in previous case–control studies is that the information on autoimmune disease in the parents was recorded independently of whether they had a child with cancer or not. We restricted the index population to those born after 1930, as children had to be living at home on 1 April 1968, when the CPR was established, in order to be traced. Still, some children may have been missed, but we have no reason to believe that these have different cancer risk than those included. Children who died before 1968 cannot be found in the CPR; for this reason the earliest start of follow-up is 1 April 1968, even for children born before to avoid selection bias.

The index parents in the present study had been hospitalized for treatment or evaluation of autoimmune disease. Even though the first hospitalization was recorded after birth for all the children with lymphoma or leukaemia observed, the disease may have been diagnosed in the parents years before birth of the child. Two points are pertinent. First, hospitalized patients may differ from non-hospitalized in various ways including the spectrum of disease and its severity. Secondly, our results relate to parental susceptibility to autoimmune disease rather than disease current at the time of index conception or birth.

Follow-up for cancer of more than 53 000 children of parents with autoimmune diseases indicates that such children are slightly more susceptible to childhood lymphoma and leukaemia than children in general. Further studies are required to confirm this association and to determine whether it is specific to certain types of autoimmune disease or to autoimmune disease present at time of conception.

### ACKNOWLEDGEMENTS

The study was supported by the Danish Cancer Society (grant no. 95 225 51). The authors thank Anne Marie Egelund Larsen at the Institute of Cancer Epidemiology, Danish Cancer Society for programming support.

### REFERENCES

Birch JM and Marsden HB (1987) A classification scheme for childhood cancer. *Int J Cancer* **40**: 620–624

Bodmer JG, Tonks S, Oza AM, Lister TA and Bodmer WF (1989) HLA-DP based resistance to Hodgkin's disease. *Lancet* **1**: 1455–1456

Brown P, Hertz H, Olsen JH, Yssing M, Scheibel E and Jensen OM (1989) Incidence of childhood cancer in Denmark 1943–1984. *Int J Epidemiol* **18**: 546–555

Buckley JD, Gilchrist GS, Ruccione K, Sather HN, Woods WG and Hammond GD (1989) Multiple sclerosis in mothers of children with acute lymphoblastic leukemia. *Leukemia* **3**: 736–739

Buckley JD, Buckley CM, Ruccione K, Sather HN, Waskerwitz MJ, Woods WG and Robison LL (1994) Epidemiological characteristics of childhood acute lymphocytic leukemia. Analysis by immunophenotype. *Leukemia* **8**: 856–864

Cid MC, Campo E, Ercilla G, Palacin A, Vilaseca J, Villalta J and Ingelmo M (1989) Immunohistochemical analysis of lymphoid and macrophage cell subsets and their immunologic activation markers in temporal arteritis. *Arthritis Rheum* **32**: 884–893

 *© 2000 Cancer Research Campaign*

Danish National Board of Health (1976) *Classification of Diseases*. Danish National Board of Health: Copenhagen
Danish National Board of Health (1998) *Cancer Incidence in Denmark 1995*. Danish National Board of Health: Copenhagen
Deodhar SD (1992) Autoimmune diseases: overview and current concepts of pathogenesis. *Clin Biochem* **25**: 181–185
Doll R (1999) The Seascale cluster: a probable explanation. *Br J Cancer* **81**: 3–5
Forgacs I (1993) Autoimmunity in ulcerative colitis. *Lancet* **341**: 602
Fox RI, Luppi M, Pisa P and Kang HI (1992) Potential role of Epstein–Barr virus in Sjögren's syndrome and rheumatoid arthritis. *J Rheumatol Suppl* **32**: 18–24
Greaves MF (1997) Aetiology of acute leukaemia. *Lancet* **349**: 344–349
Gridley G, McLaughlin JK, Ekbom A, Klareskog L, Adami H-O, Hacker DG, Hoover R and Fraumeni JF Jr (1993) Incidence of cancer among patients with rheumatoid arthritis. *J Natl Cancer Inst* **85**: 307–311
Jackson CE and Bryan WW (1998) Amyotrophic lateral sclerosis. *Semin Neurol* **18**: 27–39
Kassan SS, Thomas TL, Moutsopoulus HM, Hoover R, Kimberly RP, Budman DR, Costa J, Decker JL and Chused TM (1978) Increased risk of lymphoma in Sicca syndrome. *Ann Intern Med* **89**: 888–892
Klitz W, Aldrich CL, Fildes N, Horning SJ and Begovich AB (1994) Localization of predisposition to Hodgkin's disease in the HLA Class II region. *Am J Hum Genet* **54**: 497–505
Lakomek H-J, Plomann M, Specker C and Schwochau M (1991) Ankylosing spondylitis: an autoimmune disease? *Ann Rheum Dis* **50**: 776–781
Lucchinetti CF and Rodriguez M (1997) The controversy surrounding the pathogenesis of the multiple sclerosis lesion. *Mayo Clin Proc* **72**: 665–678
McKinney PA, Cartwright RA, Saiu J, Mann JR, Stiller CA, Draper GJ, Hartley AL, Hopton PA, Birch JM, Waterhouse J and Johnston HE (1987) The inter-regional epidemiological study of childhood cancer (IRESCC)*: a case–control study of aetiological factors in leukaemia and lymphoma. *Arch Dis Child* **62**: 279–287
Mellemkjaer L, Andersen V, Linet MS, Gridley G, Hoover R and Olsen JH (1997) Non-Hodgkin's lymphoma and other cancers among a cohort of patients with systemic lupus erythematosus. *Arthritis Rheum* **40**: 761–768
Moller DR (1997) Etiology of sarcoidosis. *Clin Chest Med* **18**: 695–706
Rothman KJ and Boice JD (1979) *Epidemiologic Analysis with a Programmable Calculator*. DHHS Publication No (NIH) 79–1649. US Government Printing Office: Washington, DC
Savage C and Lockwood CM (1990) Autoantibodies in primary systemic vasculitis. *Adv Intern Med* **35**: 73–92
Taylor GM, Robinson MD, Binchy A, Birch JM, Stevens RF, Jones PM, Carr T, Dearden S and Gokhale DA (1995) Preliminary evidence of an association between HLA-DPB1*0201 and childhood common acute lymphoblastic leukaemia supports an infectious aetiology. *Leukemia* **9**: 440–443
Taylor GM, Gokhale DA, Crowther D, Woll P, Harris M, Alexander F, Jarrett R and Cartwright RA (1996) Increased frequency of HLA-DP1*0301 in Hodgkin's disease suggests that susceptibility is HVR-sequence and subtype-associated. *Leukemia* **10**: 854–859
Taylor GM, Dearden S, Payne N, Ayres M, Gokhale DA, Birch JM, Blair V, Stevens RF, Will AM and Eden OB (1998) Evidence that an HLA-DQA1-DQB1 haplotype influences susceptibility to childhood common acute lymphoblastic leukaemia in boys provides further support for an infection-related aetiology. *Br J Cancer* **78**: 561–565
Theofilopoulos AN (1993) Molecular pathology of autoimmunity. In: *The Molecular Pathology of Autoimmune Diseases*, Bona CA, Siminovitch KA, Zanetti M and Theofilopoulos AN (eds), pp. 1–12. Chur Reading Harwood Academic: Switzerland
Till M, Rapson N and Smith PG (1979) Family studies in acute leukaemia in childhood: a possible association with autoimmune disease. *Br J Cancer* **40**: 62–71
Tonks S, Oza AM, Lister TA and Bodmer JG (1992) An international study of the association between HLA-DP and Hodgkin's disease. In: *HLA 1991 Proceedings of the Eleventh International Histocompatibility Workshop and Conference*, held in Yokohama Japan, 6–13 November, Tsuji K, Aizawa M and Sasazuki T (eds), pp. 539–544. Oxford University Press: Oxford
Wen W-Q, Shu X-O, Sellers T, Bhatia S, Lampkin B and Robison LL (1998) Family history of cancer and autoimmune disease and risk of leukemia in infancy: a report from the Children's Cancer Group (United States and Canada). *Cancer Causes Control* **9**: 161–171
Woods WG, Robison LL, Kim Y, Schuman LM, Heisel M, Smithson A, Finley J, Hutchinson R and Gibson RW (1987) Association of maternal autoimmunity with childhood acute lymphocytic leukemia (ALL). *Proc ACCR* **28**: 251
World Health Organization (1976) *ICD-O International Classification of Diseases for Oncology*. World Health Organization: Geneva

© 2000 Cancer Research Campaign 
